# Transcatheter Edge-to-Edge Repair of a Functionally Trileaflet Mitral Valve

**DOI:** 10.1016/j.jaccas.2026.109181

**Published:** 2026-07-04

**Authors:** Toheed-Fatima Ali, Hafez Golzarian, Mallory Knous, Anna Kleman, Hailey Noll, Emilie R. Scarbrough, Gerri Hempfling, Craig Imm, John Sirak, Sandeep M. Patel

**Affiliations:** a Internal Medicine Residency Program, Mercy Health—St. Rita's Medical Center, Lima, Ohio, USA; b Department of Cardiovascular Disease, University of Houston/HCA Houston Healthcare—Kingwood, Houston, Texas, USA; c Structural Heart and Intervention Center, Bon Secours Mercy Health—St. Rita's Medical Center, Lima, Ohio, USA; d Department of Anesthesiology, Bon Secours Mercy Health—St. Rita's Medical Center, Lima, Ohio, USA; e Department of Cardiothoracic Surgery, Bon Secours Mercy Health—St. Rita's Medical Center, Lima, Ohio, USA

**Keywords:** congenital, edge-to-edge repair, mitral cleft, mitral regurgitation, mitral valve, PASCAL, structural heart intervention, trileaflet mitral valve

## Abstract

**Background:**

A trileaflet configuration of the mitral valve is an uncommon anatomical finding that presents unique challenges for transcatheter repair.

**Case Summary:**

A 67-year-old man with a history of atrioventricular canal defect and prior mitral valve repair presented with symptomatic severe mitral valve insufficiency due to impaired leaflet coaptation from a cleft dividing the A2 scallop, creating a functionally trileaflet mitral valve. Given elevated surgical risk and patient preference to avoid reoperation, transcatheter edge-to-edge repair with the 2 PASCAL ACE system devices (Edwards Lifesciences) was successfully performed allowing restoration of coaptation and reduced mitral insufficiency to trace severity with a final transmitral mean gradient of 3 mm Hg.

**Discussion:**

This case highlights the adaptability of transcatheter edge-to-edge repair in unconventional mitral valve anatomy.

**Take-Home Message:**

Complex trileaflet or cleft mitral valves should not preclude transcatheter repair when guided by appropriate imaging and device strategy.

## Graphical Abstract

**Figure:**
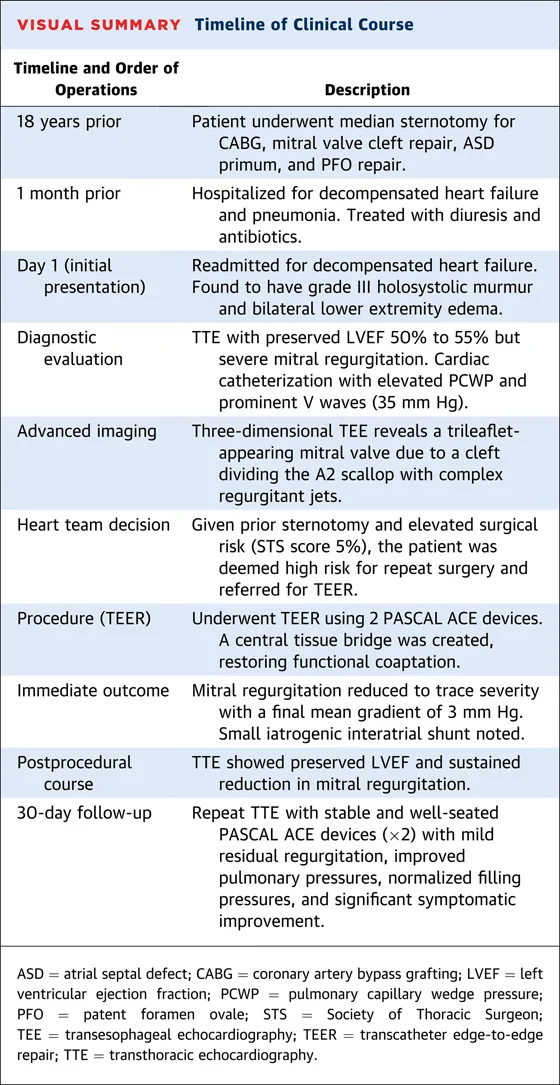

The mitral valve normally consists of anterior and posterior leaflets each with 3 scallops that coapt during systole to prevent regurgitant flow from the left ventricle to the left atrium. Structural abnormalities of mitral leaflet morphology are uncommon and most often include clefts, accessory leaflet tissue, or variations of the subvalvular apparatus.^1^^,^^2^ In rare instances, imaging may reveal a trileaflet or trileaflet-appearing configuration. Prior reports of true trileaflet mitral valves describe 3 distinct leaflet bodies separated by discrete commissures, sometimes accompanied by corresponding papillary muscle variation.^1^^,^^3^

**Take-Home Messages**

• Complex trileaflet or cleft mitral valves should not preclude transcatheter repair.
• With appropriate imaging and device strategy, transcatheter edge-to-edge repair can restore effective coaptation and achieve excellent hemodynamic outcomes.

Atrioventricular canal defects exist on a spectrum that includes complete, partial, and transitional forms, with partial atrioventricular canal defects classically associated with a primum atrial septal defect (ASD) and a cleft mitral valve. Although a cleft mitral valve does not represent a true third leaflet, it may functionally appear as an additional leaflet segment on echocardiography and create a trileaflet-appearing morphology, particularly in the setting of prior repair or distorted coaptation.^4^ Alterations in leaflet architecture can disrupt normal coaptation and contribute to significant mitral regurgitation. In patients with prior mitral valve intervention, postoperative leaflet distortion may create complex coaptation patterns that mimic additional leaflet segments and introduce unique challenges during structural repair. For this reason, accurate anatomical characterization with 3-dimensional transesophageal echocardiography (TEE) is essential when planning transcatheter intervention.

**Visual Summary:** Timeline of Clinical Course

Timeline and Order of Operations	Description
18 years prior	Patient underwent median sternotomy for CABG, mitral valve cleft repair, ASD primum, and PFO repair.
1 month prior	Hospitalized for decompensated heart failure and pneumonia. Treated with diuresis and antibiotics.
Day 1 (initial presentation)	Readmitted for decompensated heart failure. Found to have grade III holosystolic murmur and bilateral lower extremity edema.
Diagnostic evaluation	TTE with preserved LVEF 50% to 55% but severe mitral regurgitation. Cardiac catheterization with elevated PCWP and prominent V waves (35 mm Hg).
Advanced imaging	Three-dimensional TEE reveals a trileaflet-appearing mitral valve due to a cleft dividing the A2 scallop with complex regurgitant jets.
Heart team decision	Given prior sternotomy and elevated surgical risk (STS score 5%), the patient was deemed high risk for repeat surgery and referred for TEER.
Procedure (TEER)	Underwent TEER using 2 PASCAL ACE devices. A central tissue bridge was created, restoring functional coaptation.
Immediate outcome	Mitral regurgitation reduced to trace severity with a final mean gradient of 3 mm Hg. Small iatrogenic interatrial shunt noted.
Postprocedural course	TTE showed preserved LVEF and sustained reduction in mitral regurgitation.
30-day follow-up	Repeat TTE with stable and well-seated PASCAL ACE devices (×2) with mild residual regurgitation, improved pulmonary pressures, normalized filling pressures, and significant symptomatic improvement.

ASD = atrial septal defect; CABG = coronary artery bypass grafting; LVEF = left ventricular ejection fraction; PCWP = pulmonary capillary wedge pressure; PFO = patent foramen ovale; STS = Society of Thoracic Surgeon; TEE = transesophageal echocardiography; TEER = transcatheter edge-to-edge repair; TTE = transthoracic echocardiography.

Although transcatheter edge-to-edge repair (TEER) has been successfully applied across a broad range of mitral valve pathologies, reports involving trileaflet mitral valve morphology remain extremely limited.^5^ This case describes the use of the PASCAL system in a trileaflet-appearing mitral valve configuration and illustrates how this device can restore functional coaptation in an otherwise unconventional mitral valve structure.

## History of Presentation

A 67-year-old man presents with progressive dyspnea on exertion and fatigue. One month prior, he had been hospitalized for decompensated heart failure and pneumonia where he was treated with diuresis, fluid restriction, and antibiotics. Despite being discharged home on maximally tolerated guideline-directed medical therapy, he presented again 1 month later with similar complaints but now with orthopnea. On physical examination, vital signs were stable. Cardiovascular examination revealed a grade 3/6 holosystolic murmur. There was knee-high bilateral lower extremity pitting edema. The remainder of the physical examination was unremarkable.

## Past Medical History

Past medical history consisted of congenital mitral cleft, primum ASD, and coronary artery disease status post single-vessel coronary artery bypass graft, mitral valve and atrioventricular canal repair with ASD repair 18 years prior, atrial fibrillation on anticoagulation, dual pacemaker placement, heart failure with preserved ejection fraction, type II diabetes mellitus, and hyperlipidemia.

## Differential Diagnoses

The differential diagnosis for this patient's symptoms included progression of degenerative mitral regurgitation, acute coronary syndrome, decompensated heart failure, and pulmonary pathologies.

## Investigations

**Figure 1:**
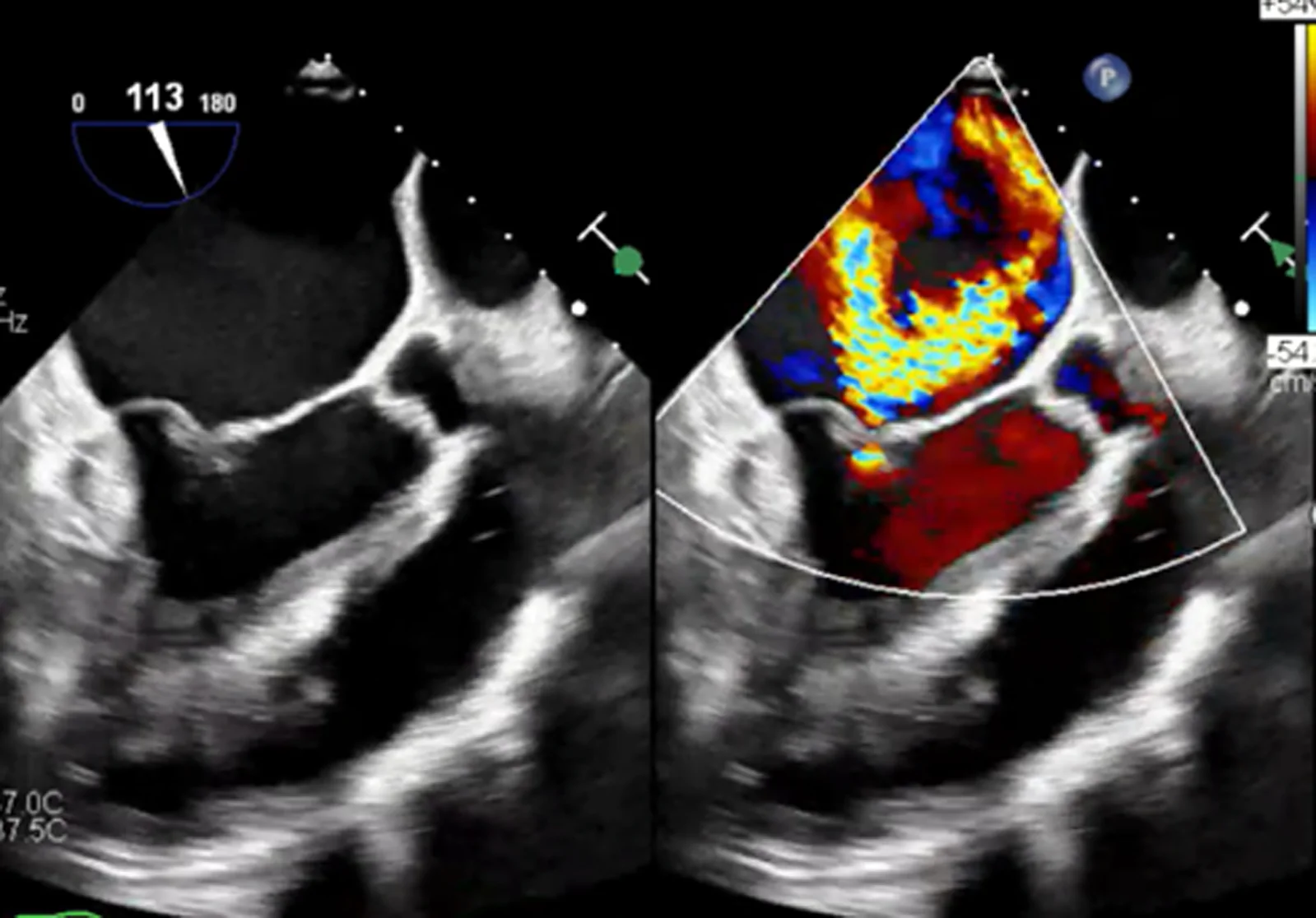
Transesophageal Echocardiography Revealing Severe Mitral Insufficiency With Multiple Eccentric Jets

Transthoracic echocardiography demonstrated preserved left ventricular systolic function with an estimated ejection fraction of 50% to 55%, moderate pulmonary hypertension with a right ventricular systolic pressure of 50 mm Hg, and severe mitral regurgitation with multiple eccentric jets (Figure 1 and Video 1).

**Figure 2:**
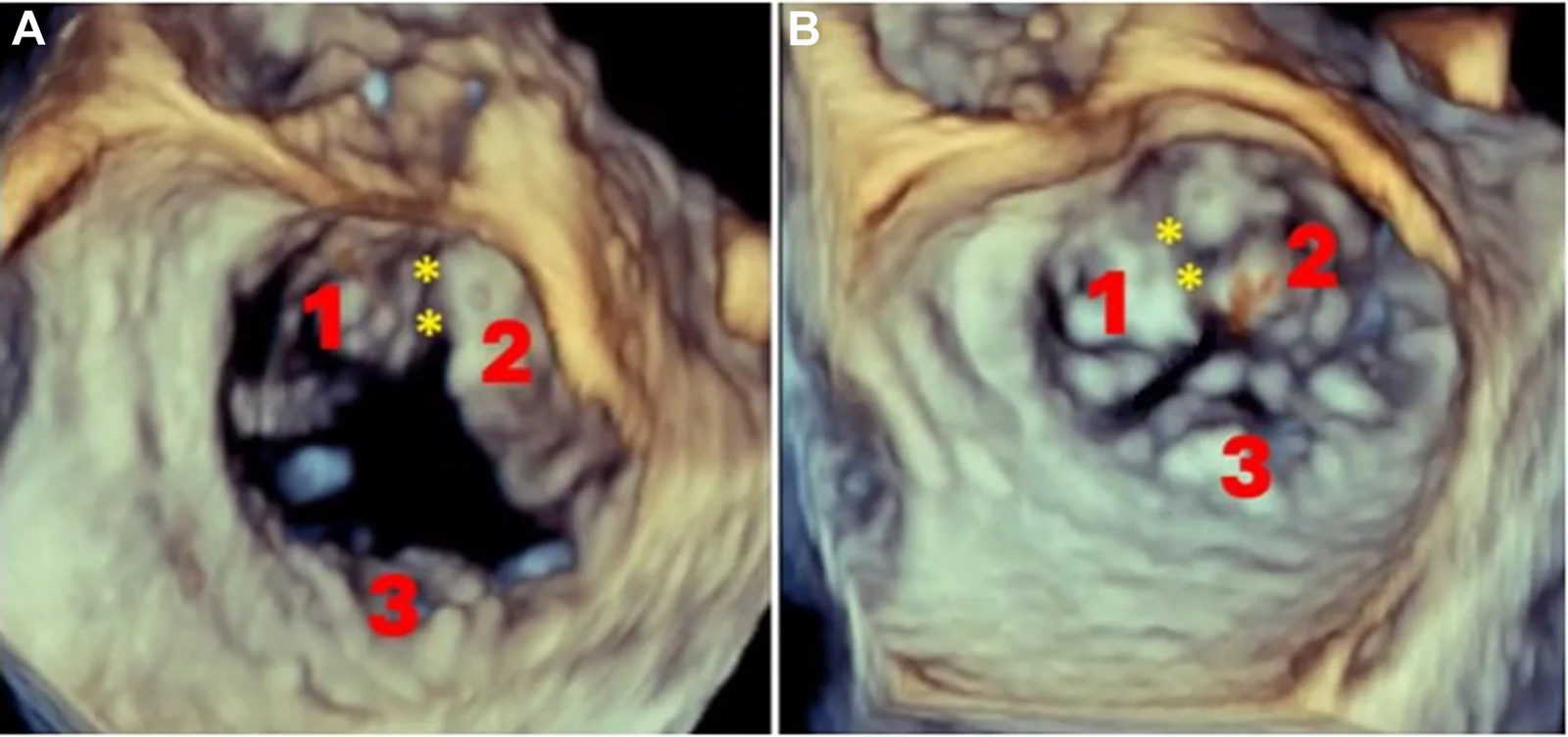
Three-Dimensional Transesophageal Echocardiographic En Face Views of the Mitral Valve Demonstrating a Trileaflet-Appearing Morphology (A) Mitral leaflet anatomy in diastole. (B) Leaflet anatomy in systole. Labels 1, 2, and 3 denote the trileaflet morphology with 3 distinct leaflets in the setting of a congenital mitral cleft, denoted by yellow asterisks.

Cardiac catheterization demonstrated patent coronary grafts, ruling out acute coronary syndrome. Hemodynamic assessment revealed pulmonary artery pressures of 40/16 mm Hg (mean 26 mm Hg), a pulmonary capillary wedge pressure of 18 mm Hg with prominent “V waves” to 35 mm Hg, a cardiac output of 5.46 L/min, a pulmonary vascular resistance of 1.46 WU consistent with postcapillary pulmonary hypertension secondary to severe mitral regurgitation, and elevated left-sided filling pressures. In conjunction with an estimated pulmonary artery systolic pressure of 50 mm Hg (by the modified Bernoulli equation) on echocardiography, and the high-risk presentation, a transcatheter approach was deemed favorable. Three-dimensional TEE revealed a trileaflet-appearing mitral valve morphology with complex regurgitant jet patterns and impaired leaflet coaptation due to a cleft dividing the A2 scallop (Figure 2).

On further review of the patient's operative report of mitral valve repair, it was noted that the patient had congenital fusion of the mitral valve to the ventricular septum, with an absent inlet portion of the ventricular septum as well as a mitral leaflet cleft, which were closed with 4-0 Prolene sutures. The anterior common bridging leaflet also had a cleft on top of the septum. During the repair, the right portion of the posterior leaflet was detached to anchor the patch into the septum for the ASD repair.

## Management (Medical/Intervention)

Given prior sternotomy, elevated surgical risk (Society of Thoracic Surgeons score 5%), and patient preference to avoid repeat surgery, the patient was evaluated by the structural heart team, which considered his previously repaired congenital atrioventricular canal anatomy, and a transcatheter approach was pursued. Under general anesthesia with TEE guidance, a superior-posterior transseptal puncture was performed approximately 4.5 to 5 cm above the mitral annulus. Using fluoroscopic and echocardiographic guidance, the PASCAL ACE system was advanced to the mitral annulus. Once positioned with paddles perpendicular to the coaptation line, the orientation of the anterior and posterior clasps was confirmed, and the first PASCAL ACE device was deployed, reducing mitral regurgitation to moderate severity (Video 2). TEE assessment showed sufficient leaflet tissue for a second device. The second PASCAL ACE device was advanced, aligned with the color jet on TEE, and positioned appropriately relative to the first device. After deploying the second device, mitral regurgitation decreased substantially. Postdeployment TEE confirmed trace mitral regurgitation, a mean gradient of 3 mm Hg, and an adequate tissue bridge. Preprocedural left atrial pressure demonstrated a mean pressure of 15 mm Hg with V waves to 22 mm Hg, which improved postprocedurally to a mean pressure of 10 mm Hg with V waves to 16 mmHg after device implantation. Device positioning created a central tissue bridge, essentially restoring functional coaptation.

## Outcome and Follow-Up

After the procedure, mitral regurgitation was reduced to trace severity with a final mean transmitral gradient of 3 mm Hg (Video 3). No pericardial effusion was observed, and a small iatrogenic interatrial shunt was noted.

Postprocedural transthoracic echocardiography demonstrated preserved left ventricular systolic function and sustained reduction in mitral regurgitation. N-terminal pro–B-type natriuretic peptide improved from 1,592 pg/mL preintervention to 905 pg/mL after TEER, supporting symptomatic and hemodynamic improvement after correction of mitral regurgitation.

At 1-month follow-up, imaging confirmed stable device position, mild residual regurgitation, improved pulmonary pressures, and normalization of intracardiac filling pressures. The patient reported significant symptomatic improvement without complications.

## Discussion

TEER has become an important treatment option for patients with severe mitral regurgitation who are at elevated surgical risk and is now routinely used in increasingly complex mitral valve anatomy.^6^ However, published experience with TEER in trileaflet or trileaflet-appearing mitral valve morphology remains extremely limited, and use of the PASCAL system in this anatomical setting has not previously been described. This case therefore represents a novel application of transcatheter repair in an unconventional mitral valve configuration.

Given the patient's prior cardiac surgery and presentation with heart failure symptoms, constrictive pericardial physiology was also considered as part of the differential diagnosis. However, imaging findings and the markedly reduced symptoms postprocedure supported the etiology being more consistent with valvular dysfunction as the most dominant mechanism of decompensation.

In this patient, a history of atrioventricular canal defect with associated cleft anterior mitral valve with prior mitral valve surgery likely contributed to altered leaflet architecture that produced a trileaflet-appearing configuration with complex coaptation patterns. Such anatomy changes the usual approach to edge-to-edge repair, which traditionally relies on approximation of the anterior and posterior leaflets to create a double orifice valve in the region of A2-P2. Careful device orientation was required to capture the appropriate leaflet segments while maintaining acceptable transmitral gradients and preserving leaflet mobility.

In our case, we noted that the anterior mitral valve leaflet cleft separated the entire leaflet almost to the annulus and allowed for functionally 3 separate leaflets that moved independently during systole and diastole. Our interventional strategy was analogous to a tricuspid edge-to-edge repair in which we start in the region of the anteroseptal commissure and move more posteriorly. Similarly, we started to place the PASCAL devices between the commissures of leaflets 2 and 3 and moved centrally (and laterally) (Figure 2).

After placement of the 2 PASCAL ACE devices, we created a central tissue bridge that effectively restored a functional pattern of coaptation that is typical of the post-TEER double-orifice morphology despite the underlying trileaflet-appearing anatomy. This strategy allowed for stable leaflet approximation with minimal residual regurgitation and acceptable transmitral gradients. The independent clasping mechanism, device elongation, and triaxial maneuverability of the PASCAL system provided additional flexibility in addressing asymmetric coaptation, minimizing regurgitation, and optimizing mitral gradients.

## Conclusions

This case highlights an unusual mitral valve configuration in which severe regurgitation arose from a trileaflet-appearing morphology after prior surgical intervention. TEER with the PASCAL system successfully restored valve competence by creating a central tissue bridge that reestablished functional coaptation. Beyond the immediate clinical outcome, this case illustrates how transcatheter repair using the PASCAL system can be adapted to address unusual leaflet architecture that would traditionally fall outside standard repair paradigms. As structural heart therapies continue to advance, recognizing and creatively managing atypical mitral valve anatomy will remain an important part of expanding the boundaries of transcatheter intervention.

### Data Availability

The data involved in this case study are available from the authors upon request.

## Funding Support and Author Disclosures

The authors have reported that they have no relationships relevant to the contents of this paper to disclose.

## Appendix

Video 1 Transesophageal Echocardiography Demonstrating Severe Mitral Regurgitation and Trileaflet Mitral Valve Morphology Before Intervention

Video 2 Intraprocedural Transesophageal Echocardiography During Deployment of the First PASCAL ACE Device

Video 3 Final Postprocedural Transesophageal Echocardiography After Deployment of 2 PASCAL ACE Devices Demonstrating Restoration of Leaflet Coaptation and Reduction in Mitral Regurgitation
